# Presence, persistence and effects of pre-treatment HIV-1 drug resistance variants detected using next generation sequencing: A Retrospective longitudinal study from rural coastal Kenya

**DOI:** 10.1371/journal.pone.0210559

**Published:** 2019-02-13

**Authors:** Amin S. Hassan, David F. Bibby, Shalton M. Mwaringa, Clara A. Agutu, Kennedy K. Ndirangu, Eduard J. Sanders, Patricia A. Cane, Jean L. Mbisa, James A. Berkley

**Affiliations:** 1 KEMRI/Wellcome Trust Research Programme, Kilifi, Kenya; 2 Virus Reference Department, Public Health England, London, United Kingdom; 3 Kilifi County Hospital, Kilifi, Kenya; 4 Centre for Tropical Medicine & Global Health, University of Oxford, Oxford, United Kingdom; University of Cincinnati College of Medicine, UNITED STATES

## Abstract

**Background:**

The epidemiology of HIV-1 drug resistance (HIVDR) determined by Sanger capillary sequencing, has been widely studied. However, much less is known about HIVDR detected using next generation sequencing (NGS) methods. We aimed to determine the presence, persistence and effect of pre-treatment HIVDR variants detected using NGS in HIV-1 infected antiretroviral treatment (ART) naïve participants from rural Coastal Kenya.

**Methods:**

In a retrospective longitudinal study, samples from HIV-1 infected participants collected prior [n = 2 time-points] and after [n = 1 time-point] ART initiation were considered. An ultra-deep amplicon-based NGS assay, calling for nucleotide variants at >2.0% frequency of viral population, was used. Suspected virologic failure (sVF) was defined as a one-off HIV-1 viral load of >1000 copies/ml whilst on ART.

**Results:**

Of the 50 eligible participants, 12 (24.0% [95% CI: 13.1–38.2]) had at least one detectable pre-treatment HIVDR variant against Protease Inhibitors (PIs, n = 6 [12%]), Nucleoside Reverse Transcriptase Inhibitors (NRTIs, n = 4 [8.0%]) and Non-NRTIs (n = 3 [6.0%]). Overall, 15 pre-treatment resistance variants were detected (frequency, range: 2.3–92.0%). A positive correlation was observed between mutation frequency and absolute load for NRTI and/or NNRTI variants (r = 0.761 [p = 0.028]), but not for PI variants (r = -0.117 [p = 0.803]). Participants with pre-treatment NRTI and/or NNRTI resistance had increased odds of sVF (OR = 6.0; 95% CI = 1.0–36.9; p = 0.054).

**Conclusions:**

Using NGS, pre-treatment resistance variants were common, though observed PI variants were unlikely transmitted, but rather probably generated *de novo*. Even when detected from a low frequency, pre-treatment NRTI and/or NNRTI resistance variants may adversely affect treatment outcomes.

## Background

Highly active antiretroviral therapy (HAART) can successfully suppress HIV replication *in vivo*. However, the expansion of HAART, as expected, has led to a parallel increase in the emergence and transmission of HIV drug resistance (HIVDR) mutations [1, 2]. Transmitted HIVDR viruses confer reduced susceptibility to, and are known to compromise the effectiveness of first-line HAART regimens [3, 4]. The prevalence of transmitted resistance is reported to be significantly higher but stable in high-income countries, and low but on the increase in low/middle income countries [1, 2]. Consequently, genotypic antiretroviral resistance testing (GART) for HIVDR is currently recommended to guide clinical care in developed settings [5], and for surveillance purposes in resource limited settings [6]. However, the inability of conventional GART using Sanger sequencing to detect resistant variants at frequencies <20% of the circulating viral quasispecies [7, 8] likely leads to an underestimation in the actual burden of HIVDR [9].

Highly sensitive methods, including codon-specific point mutation assays (allele specific PCR [AS-PCR] and Oligonucleotide Ligation Assays [OLA]) and partial/whole genome ultra-deep sequencing assays are becoming increasingly available and cost effective [10]. Compared to Sanger capillary sequencing, these ultrasensitive assays have the ability to detect HIVDR variants at low frequencies of <1% circulating quasispecies [11–13]. A systematic review and meta-analysis of HIV-1 low frequency variants studies, all from HIV-1 subtype B predominant regions and comparing AS-PCR and GART estimates, reported up to an 8-fold higher detection of some transmitted resistance variants by AS-PCR [14]. Importantly, a pooled analysis of individual data from 10 studies, all from North America and Europe, reported a significant dose-response association between presence of HIV-1 low frequency variants, particularly those conferring reduced susceptibility to NNRTIs, and increased risk of virologic failure [15], even amongst patients with high levels of adherence [16].

Only a handful of studies, all in the context of women receiving single dose Nevirapine for prevention of vertical HIV-1 transmission, and all using point mutation assays, have been reported from Africa, where the burden of HIV-1 is highest [17–20]. More recently, a study using the point mutation OLA for NNRTI variants K103N, Y181C and G190A reported a ten-fold increased risk of virological failure among treatment naïve adults initiated HAART in Kenya [21].

Hence, whilst the epidemiology of transmitted HIVDR by GART has been widely studied, much less is known about HIVDR variants detected from low frequencies using next generation sequencing (NGS) assays. The few existing studies are largely from developed settings, and applied codon-specific point mutation assays particularly targeting NRTI-associated M184V and NNRTI-associated K103N, Y181C and G190A mutations. To date, about 93 transmitted HIVDR mutations conferring reduced susceptibility to NRTIs (n = 34), NNRTIs (n = 19) and protease inhibitors (PIs, n = 40) have been identified and are recommended for surveillance by the WHO [22].

We aimed to describe the presence, persistence and effect of HIVDR variants detected using an ultra-deep amplicon-based NGS assay in chronically HIV-1 infected ART-naïve individuals from a rural HIV clinic in Coastal Kenya, an HIV-1 non-B subtype predominant region.

## Methods

### Study site

Participants were recruited from the HIV clinic at Kilifi County Hospital, a public health facility located in rural Coastal Kenya. The hospital has a catchment population of an estimated 260,000 people, majority of whom practice subsistence farming as the mainstay socio-economic activity. HIV care and treatment services in the hospital are provided according to national guidelines [23]. In brief and at the time of the study, HAART eligibility included individuals with WHO clinical staging III/IV regardless of CD4 T-cell count, or individuals with CD4 T-cell count of <350 cells/mm^3^ regardless of WHO clinical staging, or both. Standard first-line regimen comprised two nucleoside reverse transcriptase inhibitors (NRTI) and a non-nucleoside reverse transcriptase inhibitor (NNRTI). Individuals failing first-line regimen were switched to a second line regimen comprising two NRTIs and a boosted protease inhibitor (PI). Previous work suggests that this is largely a non-B subtype region, with pre-dominance of HIV-1 subtypes A1, C and D [24].

### Study design

A retrospective longitudinal study design, nested in a facility-based HIV surveillance cohort was adopted. In brief, HIV-infected, newly diagnosed individuals enrolling for care at the clinic were recruited and followed up between 2008 and 2013. All participants reported no previous exposure to antiretroviral therapy, including prevention of mother to child transmission (pMTCT) interventions, at enrolment into care. Remnant blood samples from routine diagnostic investigations were used to obtain plasma, which was archived at -80°C.

For the purpose of this study, ART-naïve individuals with at least three longitudinal archived samples that met the following criteria were considered: i) an enrolment sample (collected at enrolment into care and at least 6 months prior to treatment initiation); ii) an ART baseline sample (collected at most 3 months prior to treatment initiation); and iii) an on-treatment sample (collected at least 6 months after treatment initiation).

### Laboratory methods

#### i) RNA extraction, PCR amplification and next generation sequencing

These have been previously described in detail elsewhere [25, 26]. In brief, HIV-1 viral RNA was manually extracted using the QIAamp UltraSens protocol (QIAGEN, Hilden Germany), with a starting volume of 200 μL of plasma and eluted in 60 μL Buffer AVE.

An amplicon-based approach, targeting the *pol* subgenomic region of HIV-1 containing protease and part of reverse transcriptase gene (between positions 2250–3550 of the reference HxB2 sequence) was used. The fragment was amplified by single-step reverse transcription (RT)-PCR followed by a nested PCR reaction. For samples that failed to amplify, a second repeat PCR was attempted. Archived plasma was retrieved and a repeat RNA extraction done on samples that failed the repeat PCR. Then, a third repeat PCR attempt was done. Results from the repeat extraction and PCR attempts are summarised in the supplementary material (S1 Fig).

Nested PCR products were quantified with the Qubit 2.0 fluorometer using the Quant-iT assay kit (Life technologies). One ng/μl of the amplified DNA was used for library preparation with the Nextera XT DNA sample prep kit (Illumina).

NGS was performed on the MiSeq system (Illumina). In brief, samples concentrations were validated and standards added to the consolidation plate. The consolidation plate was then replicated and quantified to two dilution plates of 1ng/ul and 0.2 ng/ul respectively. The second dilution plate was further replicated into a Nextera XT library preparation plate where adapter sequences are attached for tagmentation and PCR amplification. The amplified plate underwent normalization and fragmentation into a pooled amplicon library (PAL) plate, which was quantified and further reconstituted into the diluted amplicon library (DAL). DAL was then loaded and attached onto a HiSeq flow cell and reagents, which were then subsequently loaded onto a MiSeq catridge for sequencing. Short read fragments were generated in a standard FASTQ format

#### ii) Bioinformatics and sequence handling

This was done using an automated computational pipeline developed in-house using Python and C++ and run through a workflow in Galaxy. In brief, the raw FASTQ data were taken through quality trims and filters. A subset of the reads from each FASTQ file was compared to a local database of HIV reference sequences using BLAST in order to identify an optimum reference sequence. Reference mapping was then performed using BWA-MEM (version 0.7.5). The resulting files were converted into BAM format using SAMTools in preparation for the in-house developed QuasiBAM software, which generated consensus sequences with user-specified variant thresholds for the inclusion of minority nucleotides. For the purpose of this study, HIV-1 drug resistance mutations were called if they had nucleotide variants occurring at frequencies >2.0% of the sample quasispecies. While this threshold has been shown to yield better reproducibility in the detection of HIV-1 drug resistance variants generated from NGS assays [26], it may still not represent an accurate estimate of the frequency of circulating variants, especially in samples with low viral loads, as the actual number of viral templates input used in the sequencing reaction was not empirically determined.

#### iii) HIV-1 subtyping and drug resistance interpretation

Consensus sequences were submitted to the REGA HIV-1 subtyping tool v3.0 [27], and cross referenced with the subtype classification using Evolutionary Algorithm (SCUEAL) tool on Datamonkey [28]. Indeterminate or discordant sequences were further submitted to the jumping profile hidden Markov Model (jphMM) tool [29] for determination of recombinants.

Consensus sequences were submitted to the Stanford HIVDR database to identify resistance mutations and interpret genotypic susceptibility [30]. Transmitted and acquired HIVDR mutations were confirmed using the list for surveillance of transmitted drug resistance and the updated list of major resistance mutations from the HIV Drug Resistance Database respectively [22, 31].

#### iv) HIV-1 viral load quantification

HIV-1 RNA viral load quantification was done using an in-house real time qPCR assay with a detection threshold of 400 copies/ml. In brief, 25ul reactions of 12.5 ul RNA extract and 12.5 ul mastermix of the QIAGEN QuantiTect kit, probes and a set of integrase forward and reverse primers were used. Quantification was done on the ABI Prism 7500 real time PCR machine and results interpreted based on a standard curve with calibrated in-house standards. A one-off viral load was used to determine suspected virologic failure (sVF), defined as HIV-1 RNA viral load of >1000 copies/ml whilst on HAART.

### Data analysis

Data analysis was done using Stata/SE version 13.1 (Stata corp. TX). The overall prevalence of pre-treatment HIVDR (95% confidence intervals, CI) was determined as a percentage of participants with at least one detectable resistance variant at >2.0% frequency in the two pre-treatment time-points over the total number of participants included in the analysis. The prevalence estimate was further stratified by time points and antiretroviral drug classes. In addition, the overall and drug-class specific relationship between mutational frequencies and absolute mutational load (as a product of mutational frequencies and viral load) was assessed using Pearson’s correlation coefficients. Logistic regression was used to determine the effect of pre-treatment HIVDR on sVF.

### Ethical considerations

Science and Ethics approvals were granted by the National Scientific Steering Committee and the Ethics and Review Committee of the Kenya Medical Research Institute (SSC No. 1341). All participants gave written informed consent to participate in the study. All pre-treatment consensus HIV-1 *pol* sequence fasta files are available from GenBank (Accession numbers MH575294-MH575373).

## Results

### Characteristics of study participants

Overall, 50 participants met our eligibility criteria (females, n = 36 [72.0%]; median age at ART initiation, 34.7 [IQR, 28.9–42.0] years). Most of the participants had HIV-1 subtype A1 (n = 32 [64.0%]) (Table 1).

**Table 1:** Characteristics of individuals enrolled for HIV-1 care and included in the analyses from rural Coastal Kenya (N = 50).

Characteristics		Male (n = 14)	Female (n = 36)	Total (n = 50)
HIV-1 subtype	A1	10 [71.4]	22 [61.1]	32 [64.0]
	C	0 [0.0]	4 [11.1]	4 [8.0]
	D	2 [14.3]	2 [5.6]	4 [8.0]
	Others	2 [14.3]	8 [22.2]	10 [20.0]
Age at ART initiation (years)	Median	37.9	33.9	34.7
	[IQR]	[21.1–43.0]	[29.4–41.7]	[28.9–42.0]
Age group at ART initiation (years)	≤ 24	4 [28.6]	4 [11.1]	8 [16.0]
	25–34	1 [7.1]	17 [47.2]	18 [36.0]
	≥ 35	9 [64.3]	15 [41.7]	24 [48.0]
Year of ART initiation	2009	4 [28.6]	6 [16.7]	10 [20.0]
	2010	6 [42.9]	14 [38.9]	20 [40.0]
	2011	3 [21.4]	13 [36.1]	16 [32.0]
	2012	1 [7.1]	3 [8.3]	4 [8.0]
Initial ART regimen	AZT/3TC/EFV	0 [0.0]	1 [2.8]	1 [2.0]
	AZT/3TC/NVP	11 [78.6]	31 [86.1]	42 [84.0]
	D4T/3TC/NVP	3 [21.4]	3 [8.3]	6 [12.0]
	TDF/3TC/EFV	0 [0.0]	1 [2.8]	1 [2.0]

Abbreviations: IQR (Interquartile range); ART (Antiretroviral therapy); AZT (Zidovudine); 3TC (Lamivudine); EFV (Efavirenz); NVP (Nevirapine); D4T (Stavudine); TDF (Tenofovir); BMI (Body Mass Index) and Others (B [n = 1]; G [n = 1]; A1,C recombinant [n = 2]; A1,D recombinant [n = 4]; C,D recombinant [n = 2]).

Three longitudinal samples were retrieved from each participant at a median duration of 11.6 (min/max: 6.4–42.2) months prior to ART initiation, 1.2 (0.0–2.9) months prior to ART initiation and 21.3 (6.1–45.9) months after ART initiation, respectively. The median CD4 T-cell count dropped from the first time-point to the second-time point prior to ART initiation but increased after ART initiation as expected. Similarly, the median BMI was relatively low in the first and second time points prior to ART initiation but increased after treatment initiation (Table 2).

**Table 2:** Distribution of clinical parameters amongst eligible participants over the three time points included in the analysis from rural Coastal Kenya (N = 50).

Characteristics		Time point 1 (Pre-ART)	Time point 2 (At ART)	Time point 3 (After ART)
Duration to/from ART initiation (months)	Median	11.6	1.2	21.3
	[min/max]	[6.4–42.2]	[0.0–2.9]	[6.1–45.9]
CD4 T-cell count	Median	348	214	397
	[min/max]	[16–1076]	[0–1106]	[65–1150]
CD4 T-cell count categories	< 200	9 [18.0]	17 [34.0]	1 [2.0]
	200–350	16 [32.0]	17 [34.0]	19 [38.0]
	>350	25 [50.0]	6 [12.0]	25 [50.0]
	Missing	0 [0.0]	10 [20.0]	5 [10.0]
BMI (Kg/m^2^)	Median	19.2	19.7	21.1
	[min/max]	[10.4–30.2]	[13.9–37.5]	[10.0–35.9]
BMI categories (Kg/m^2^)	< 18.5	17 [34.0]	15 [30.0]	12 [24.0]
	> 18.5	31 [62.0]	26 [52.0]	34 [68.0]
	Missing	2 [4.0]	9 [18.0]	4 [8.0]

Abbreviations: ART (antiretroviral therapy); min/mx (minimum/maximum); BMI (Body Mass Index).

### Presence of HIV-1 pre-treatment drug resistance variants

**Fig 1:**
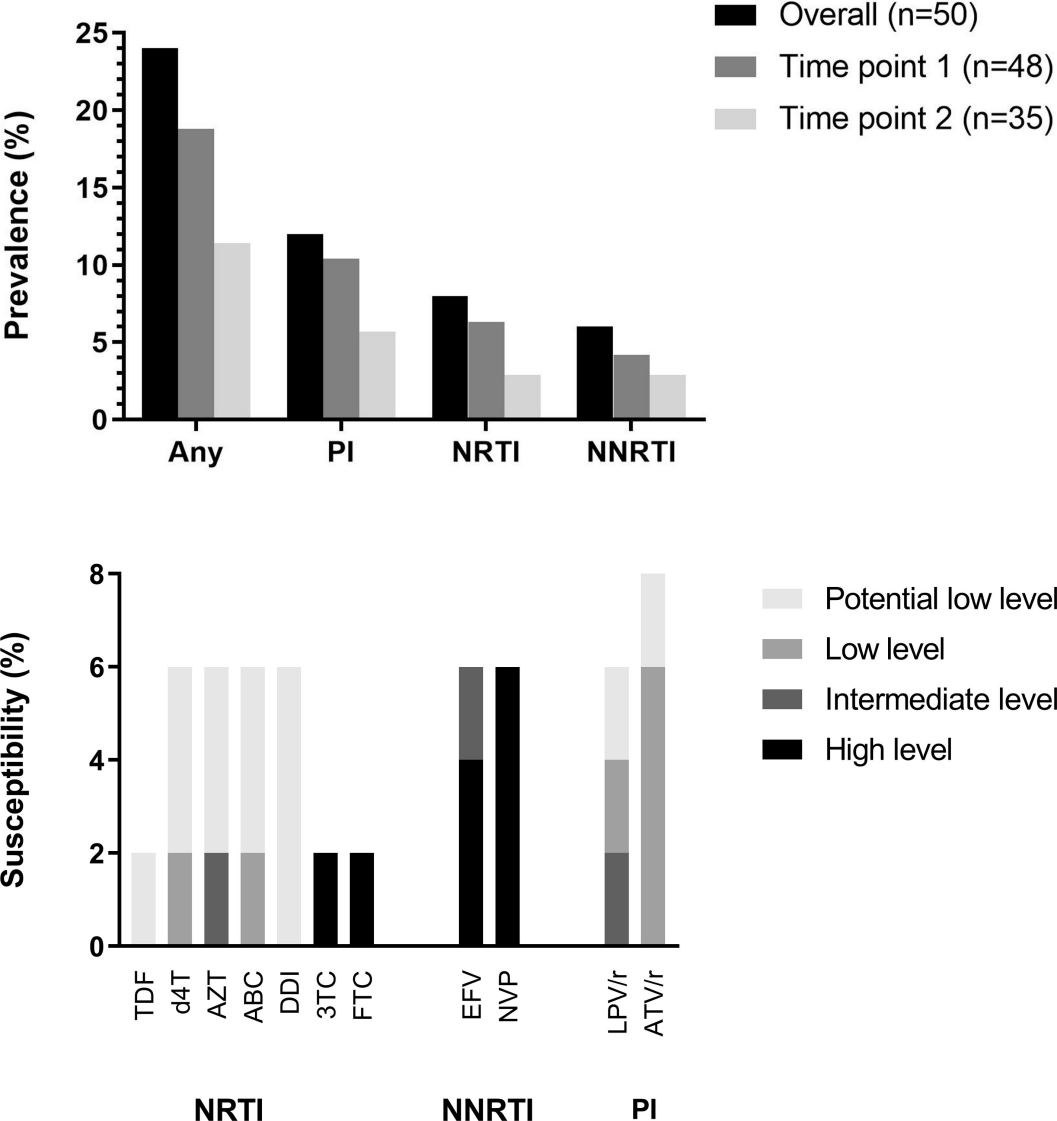
Bar graphs showing overall distribution of [a] HIV-1 drug resistance (HIVDR) variants by time points and drug classes, and [b] genotypic susceptibility of pre-treatment HIVDR variants detected by next generation sequencing from HIV infected antiretroviral-naïve individuals in a rural clinic in Coastal Kenya (n = 50)^$^.

We were able to successfully amplify and sequence at least one sample from all the 50 participants, either from the first (n = 48) or the second (n = 35) time points prior to ART initiation. There were no significant differences in successful amplification of time-point 2 samples by sampling year; 65.6% of samples collected in 2009/10 successfully amplified, compared to 77.8% collected in 2011/12 (p = 0.368). The available 83 sequences, each covering about 1300 nucleotides of the HIV-1 *pol* gene, had a mean depth of 49,820 (min/max: 1,782–74,603) reads per nucleotide position. Overall, 12 (24.0% [95% CI: 13.1–38.2]) participants had at least one detectable pre-treatment HIVDR variant against PIs (n = 6 [12.0%]), NRTIs (n = 4 [8.0%]) and NNRTIs (n = 3 [6.0%]). Exclusively, 3 (6.0%) had majority (frequency, >20%) resistance variants whilst 10 (20.0%) had minority (frequency, >2% and <20%) resistance variants. Overall, the prevalence of any pre-treatment HIVDR tended to drop from the first time-point (n = 9 [18.8%]) to the second time-point (n = 4 [11.4%]). This was also consistent within drug classes (Fig 1[A]). High-level resistance was observed against Nevirapine, Efavirenz, Lamivudine and Emtricitabine, whilst intermediate level resistance was observed against Zidovudine and Efavirenz (Fig 1[B]).

Overall, 15 mutations (frequency, range: 2.3–92.0%) were detected conferring reduced susceptibility to PIs (n = 7), NRTIs (n = 4) and NNRTIs (n = 4), with the most common being the PIs-associated D30N (n = 2) and the NNRTIs-associated G190A (n = 2). Only one participant had dual class resistance with mutations against both NRTIs (D67G) and NNRTIs (V106IM). Similarly, only one participant had more than one resistance variant within a single drug class (NNRTIs; K103N and G190A) (Table 3).

**Table 3:** Characteristics and distribution of participants with HIV-1 pre-treatment drug resistance variants detected by next generation sequencing in a rural HIV clinic in Coastal Kenya (N = 50).

Participant Identifier	Time point (duration)*	Subtype	Viral load** (Log copies/ml)	PI mutation (frequency, %)	NRTI mutations (frequency, %)	NNRTI mutations (frequency, %)
ARVR_0081	1	A1, D	4.435	M46I (5.0)	-	-
	2 (6.4)					
ARVR_0152	1	A1	4.932	-	K70R (4.0%)	
	2 (6.7)					
ARVR_0269	1	A1	4.531	D30N (9.9)	-	-
	2 (20.0)					
ARVR_0399	1	C	5.597	-	D67G (14.4)	V106M (2.5)
	2 (6.1)	C		-	-	-
ARVR_0529	1	C, D		-	-	-
	2 (24.1)	C, D	5.813	-	-	K103N (13.1) **G190A (80.3)**
ARVR_0685	1	C	3.865	**V32I (92.0)**	-	-
	2 (12.8)					
ARVR_0720	1					
	2 (6.0)	A2, D	5.398	-	M184I (16.0)	-
ARVR_1005	1	CRF01 AE		-	-	-
	2 (11.2)	CRF01 AE	6.269	I54S (3.6)	-	-
ARVR_1101	1	A1, C	3.783	V82A (4.2)	-	-
	2 (9.3)	A1, C	4.049	I47V (2.3)	-	-
ARVR_1255	1	C	5.063	-	-	**G190A (75.4)**
	2 (8.5)	C		-	-	-
ARVR_1363	1	A1	5.443	D30N (2.9)	-	-
	2 (19.5)					
ARVR_1413	1	A1	5.044		F77L (6.7)	-
	2 (6.9)					

*Duration between the first and the second sampling points in months

^**^HIV-1 RNA viral load quantification done only for samples with detectable pre-treatment resistance; shaded rows show samples not successfully amplified.

There were no significant differences in the presence of pre-treatment HIVDR by HIV-1 subtype; 18.8% of individuals with HIV-1 subtype A1 had pre-treatment HIVDR, compared to 33.3% of those with HIV-1 non-A1 subtypes (p = 0.246).

### Persistence of pre-treatment HIV-1 drug resistance variants

**Fig 2:**
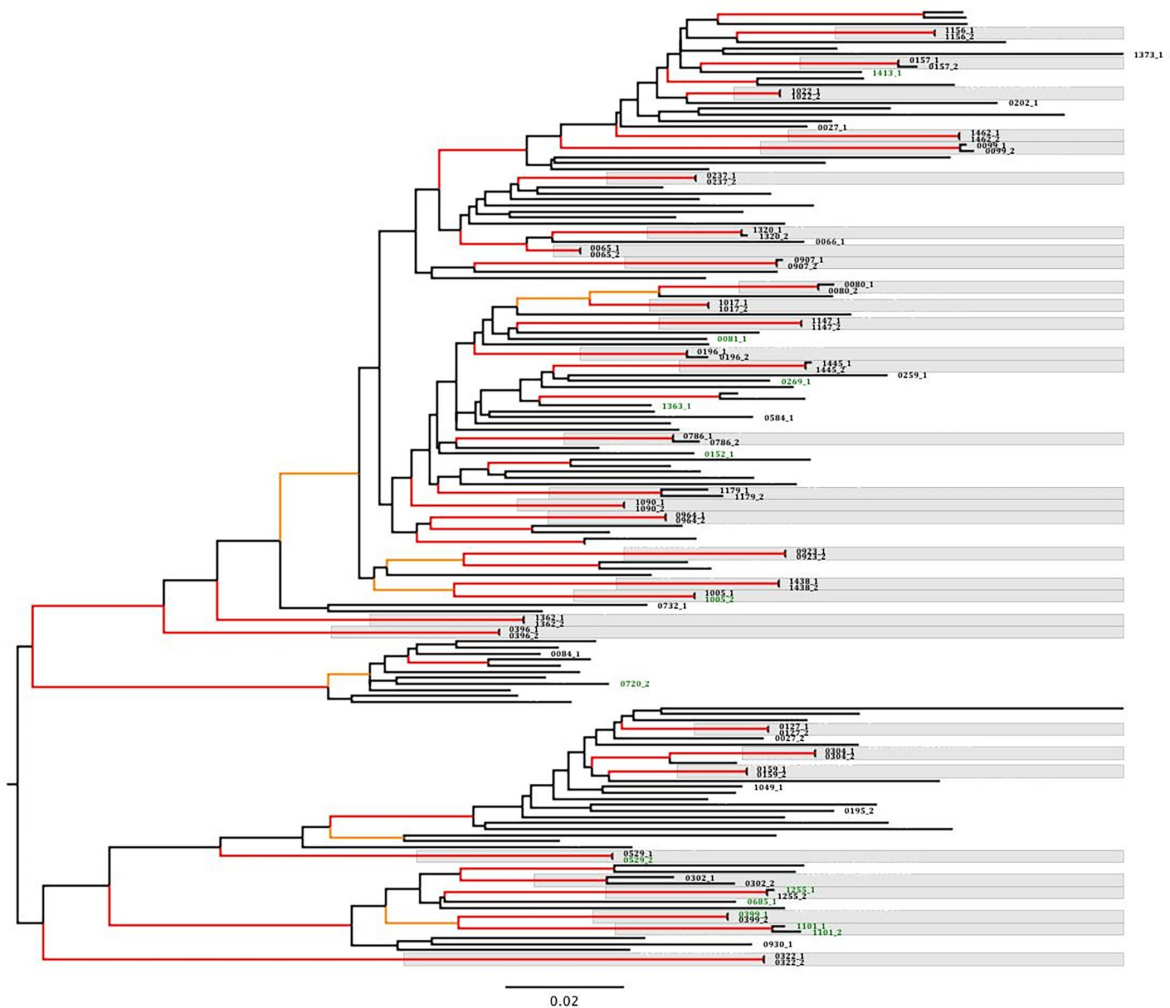
Phylogenetic tree of 83 pre-treatment HIV-1 consensus sequences collected at the first and/or second time points from HAART naïve participants (N = 50). Phylogenetic tree constructed from consensus fasta files by Maximum-likelihood estimation using the GTR model of nucleotide substitution with gamma distributed rate heterogeneity and approximate likelihood ratio test Shimodaira-hasegawa (aLRT-SH) branch support assessment. Local ‘reference’ sequences (taxa not labelled, n = 78) included. Branches leading to nodes with aLRT-SH support of >0.90 and >0.95 are coloured orange and red respectively. Individual sequences are labeled in bold letters ending with 1 (first time point) or 2 (second time point). Sequences with at least one surveillance HIV drug resistance mutation detected at >2% frequency by next generation sequencing are coloured in green. Sequence pairs from the same participant shaded in grey.

Of the 50 participants included in the study, we were able to successfully amplify and sequence pre-treatment longitudinal pairs (time points one and two) from 32 (64.0%) participants. In a phylogenetic tree, all the paired individual sequences clustered together (Fig 2). Of these, five had at least one detectable HIVDR variant in either (or both) of the two pre-treatment time points.

The first participant had dual class resistance, with mutations both to NRTIs (D67G [14.4%]) and NNRTIs (V106IM [2.5%]) at the first time-point, which were no longer detectable at the second-time point (duration, 6.1 months). The second participant had one PI mutation (V82A [4.2%]) at the first time-point, which was replaced by another PI mutation (I47V [2.3%]) at the second-time point (duration, 9.3 months). The third participant had no detectable low frequency variant at the first time-point but developed a PI mutation (I54S [3.6%]) at the second time-point (duration, 11.2 months). The fourth participant had a detectable NNRTI resistance mutation (G190A [75.4%]) at the first time-point, which was no longer detectable at the second time-point (duration, 8.5 months). The fifth participant had no detectable mutations at the first time-point, but two NNRTI mutations (K103N [13.1%] and G190A [80.3%]) were detected at the second time-point (duration, 24.1 months), possibly resulting from unreported antiretroviral exposure (Table 3).

**Fig 3:**
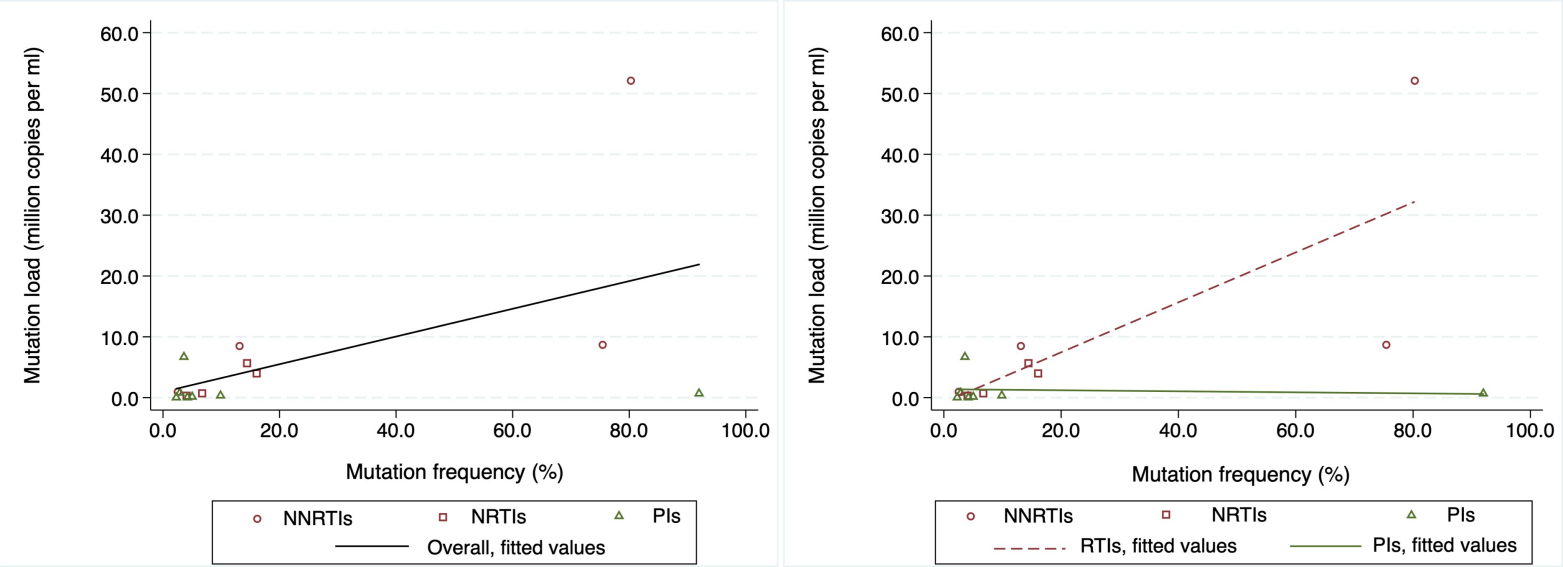
Scatter graphs illustrating the distribution and relationship between HIV-1 low frequency pre-treatment drug resistance mutation frequency and mutation load amongst [a] overall number of mutations, and [b] drug-class specific mutations observed from a HIV clinic in rural Kenya (N = 15)*.

Overall, a positive correlation was observed between mutation frequency and absolute mutation load (Pearson’s correlation, r = 0.549 [p = 0.034]) (Fig 3[A]). When stratified by drug classes, a positive correlation was also observed between mutation frequency and absolute mutation load for NRTI and/or NNRTI mutations (r = 0.761 [p = 0.028]), but not for PI mutations (r = -0.117 [p = 0.803]) (Fig 3[B]).

### Effects of HIV-1 pre-treatment drug resistance variants

Of the 50 eligible participants, we were able to successfully extract and quantify HIV-1 RNA from 48 samples from the third (after ART initiation) time point. Of these, 9 (18.8% [95% CI: 8.9–32.6]) had sVF, with a median (log 10) viral load of 4.946 (IQR: 3.937–5.196) copies/ml (Table 4).

**Table 4:** Distribution and characteristics of participants with suspected virologic failure and HIV-1 acquired resistance variants from a HIV clinic in rural coastal Kenya (n = 48) *.

Participants identifier	Baseline subtype	Baseline resistance mutations			Initial HAART regimen	Duration on HAART	Viral load (log cpm)	Acquired resistance mutations		
		PIs (%)	NRTIs (%)	NNRTIs (%)				PIs (%)	NRTIs (%)	NNRTIs (%)
ARVR_0127	D	-	-	-	AZT/3TC/NVP	24.8	5.282	-	-	-
ARVR_0159	D	-	-	-	AZT/3TC/NVP	37.1	3.937	-	-	**K103N (99.4)**
ARVR_0202	A1	-	-	-	AZT/3TC/NVP	31.0	3.360			
ARVR_0259	A1	-	-	-	D4T/3TC/NVP	13.5	6.870	-	**D67N (98.6)** T69N (2.9) **K70R (98.9)** **M184V (99.5)** **T215F (91.7)** **K219E (96.9)**	**Y181C (99.7)**
ARVR_0399	C	-	D67G (14.4)	V106IM (2.5)	AZT/3TC/NVP	30.6	3.090			
ARVR__1090	A1	-	-	-	AZT/3TC/NVP	13.1	5.196	-	**M184V (99.5)**	**K101E (50.8)** **K103NS (22.9)** **G190A (99.7)**
ARVR_1255	C	-	-	**G190A (75.4)**	AZT/3TC/NVP	23.4	4.945	-	-	-
ARVR_1362	A1	-	-	-	AZT/3TC/NVP	21.2	5.480	-	-	-
ARVR_0529	C, D	-	-	K103N (13.1) **G190A (80.3)**	AZT/3TC/NVP	19.9	3.607			

*Two samples not quantified due to very low volumes; Shaded cells (samples not successfully amplified and/or next generation sequenced).

Of the 12 and 36 participants with and without any pre-treatment HIVDR variants, 3 (25.0%) and 6 (16.7%) had sVF, respectively. Similarly, of the 6 and 42 participants with and without a pre-treatment NRTI and/or NNRTI variant, 3 (50.0%) and 6 (14.3%) had sVF, respectively. All participants with a pre-treatment PI mutation achieved virologic suppression.

Overall, presence of any pre-treatment HIVDR did not have an effect on sVF (OR [95% CI], p-value: 1.7 [0.3–8.2], p = 0.527). However, participants with pre-treatment NRTI and/or NNRTI resistance had a modest six-fold increased odds of sVF, compared to those without pre-treatment NRTI and/or NNRTI resistance (OR [95% CI], p = value: 6.0 [1.0–36.9], p = 0.054).

We were able to amplify and sequence six of the nine samples with sVF. Of these, 3 (50.0%) had acquired at least one HIVDR variant, with two having dual class resistance mutations to NRTIs and NNRTIs (Table 4). Amongst those with detectable acquired resistance, all had intermediate to high-level resistance to Nevirapine and Efavirenz. No PI-associated mutations were detected, with all the participants remaining fully susceptible to recommended second line PI options.

## Discussion

Our data from rural Coastal Kenya suggest that about one in four ART naive patients with chronic HIV infection have at least one surveillance HIVDR mutation detected at >2% frequencies using highly sensitive NGS methods. This represents an overall 10–20 fold increased detection of resistance variants at lower thresholds, when compared to recent estimates from the same setting but determined by conventional GART using Sanger capillary sequencing [32]. There was no significant difference between estimates from the two studies, when compared at >20% threshold, confirming that the increased fold difference is attributed to detection of additional low frequency variants.

Given the wide range of pre-treatment mutations observed, even within drug classes, use of codon specific point mutation assays, mostly targeting amino acid positions K103, Y181, G190 and M184 would have significantly underestimated the overall prevalence of surveillance HIVDR mutations determined by NGS in this setting. Indeed, recent OLA data for NNRTI variants K103N, Y181C and G190A from a Kenyan study report pre-treatment HIVDR estimates of 3.9% amongst ART-naïve individuals [21], which is significantly underestimated when compared to this study, and may warrant consideration for the screening for all WHO recommended drug resistance mutations by NGS, at least for surveillance purposes. However, given resource constraints and technical capacity challenges, it is also important to acknowledge that OLA may present a more feasible option for routine diagnostics in resource limited settings, compared to NGS platforms.

The observed decline in the prevalence of detectable pre-treatment resistance variants from the first time-point to the second time-point, especially within reverse transcriptase inhibitors variants, is likely attributed to decreased viral fitness of transmitted variants and the subsequent replacement by wild type virus with better replicative capacity [33, 34]. Indeed, cross sectional GART data from treatment experienced patients in this setting report prevalent acquired mutations at codon positions M184, K103, Y181 and G190 [35], which would be expected to be propagated to new infections. Given that the sampled population is largely chronic, presence of the relatively stable and persistent NNRTI mutations G190A and K103N, together with the thymidine analogue mutations K70R and D67G, and the absence of the less stable M184V and Y181C mutations, which have high fitness costs and may have decayed to very low levels below the limit of detection of our assay, is therefore not surprising [33, 34].

High levels of pre-treatment PI mutations compared to NRTI and NNRTI mutations were observed, which is consistent with data from some developed [13, 36] and developing settings [37]. Whilst these may, in part, be attributed to the high levels of PI coverage in developed settings, the same may not apply in our setting where PIs were only recommended as a second line option and coverage was low, at less than 5%, at the time of the study. Most of the observed PI mutations were sporadic in appearance, occurred in isolation and at very low frequencies of <5%. In addition, whilst there was a positive correlation between NRTI and NNRTI mutation frequency and absolute mutation load, no correlation was observed amongst PI mutations. These data therefore suggest that the observed PI mutations, at least in our context, are likely to have developed *de novo* from viral diversification, and strengthens the suggestion that absolute mutation load may likely be a better indicator for identifying surveillance HIVDR at low frequencies [15, 38].

A good proportion of our pre-treatment samples failed to successfully amplify, despite multiple attempts at RNA extraction and PCR amplification. Given that almost all the samples that failed to amplify were from the second time point, it is likely that these participants may have been initiated on HAART elsewhere and after our time point 1 visit, thus achieved virologic suppression by the time they were returning to our clinic for the second time point sampling.

The observed levels of sVF are consistent with literature from low- and middle-income settings [39] and comparable to what has been previously reported in this setting [35]. Despite the low numbers, participants with a pre-treatment NRTI or NNRTI mutation demonstrated a six-fold increased odds of sVF. However, and in spite of the presence of pre-treatment NRTI or NNRTI mutations in some participants, a significant drop in their viral load was observed, with some achieving virologic suppression after treatment initiation, which may be attributed to the multiple positive effects of combination antiretroviral therapy. Thus, it remains unclear if those with sVF were a result of partial suppression with on-going viral replication, or complete suppression followed by virologic rebound secondary to acquired resistance.

Unlike the pre-treatment variants, most of the acquired resistance mutations out-competed wild type virus and constituted more than 90% of the circulating quasispecies, which may be a consequence of selection by antiretroviral therapy. Of particular interest is the absence of the G190A mutation after treatment initiation in a participant who had this mutation at enrolment into care. Noteworthy is that the mutation had undergone viral decay and was replaced with wild type virus even before initiation of antiretroviral therapy, which may explain its absence after treatment initiation. As we did not quantify viral templates inputted into each RT reaction, it is also possible that the G190A variant was present as a minority variant and was detected at a higher frequency due to differential input of viral templates. Thus, the resulting sVF may therefore be explained by non-adhrence to HAART. This finding is consistent with data from other studies reporting absence of most variants detected prior to treatment initiation during virologic failure [40] and warrant further investigations on the re-emergence and potential effects of transmitted variants that revert to the wild type and are undetectable at treatment initiation, even at low frequencies, on long term treatment outcomes.

Of concern, however, is the presence of dual class resistance mutations conferring reduced susceptibility to both NRTIs and NNRTIs in two participants around a year after starting therapy, with one of these having multiple thymidine analogue mutations D67N, K70R, T215F, K219E, and the multidrug resistance T69N variant. These participants did not have any pre-treatment resistance, suggesting that the acquired mutations are likely to be due to sub-optimal adherence [41, 42], underpinning the vital role of adherence support in ART programs in resource limited settings, especially in light of recent policy move to put all HIV-infected individuals on HAART [43]. The expected absence of PI mutations after first line non-PI regimen, even at low frequencies, complements evidence supporting continued recommendation of PI-based second line regimen in this setting.

Our study had a few inherent limitations. Firstly, we had a small sample size with few longitudinal time points, which results to wide uncertainties in our observations. As such, it is likely that the observed sVF may in part be attributed to pre-treatment mutations at frequencies >20% or acquired mutations resulting from sub-optimal adherence. Secondly, we studied a largely chronic population with long-standing HIV infection. Hence, it is likely that we have missed out less stable transmitted variants that may have been out-competed by wild type virus by the time of study enrolment [33, 44], which may have resulted in an underestimation of the prevalence of pre-treatment resistance in this population. Lastly, we used a conservative threshold of >2% frequency to effectively exclude random mutations arising from errors related with reverse transcription, PCR amplification and sequencing artefacts [45]. However, the presence of genuine pre-treatment HIVDR variants occurring at <2% frequency cannot be ruled out.

In conclusion, and limitations notwithstanding, our pilot data from a rural HIV clinic in Coastal Kenya suggest significantly higher levels of pre-treatment HIVDR detected by a more sensitive NGS method. Results from this non-subtype B predominant region with low PI coverage suggest that the pre-treatment PI variants observed are unlikely transmitted, but rather probably *de novo* generated. Whilst the study was not powered to determine an independent effect of pre-treatment low frequency variants on treatment outcomes, these data point towards a possible deleterious effect of NRTI and/or NNRTI mutations detected by NGS from a low frequency on treatment outcomes, though their pathways and cut-off thresholds of clinical significance remain unknown. Bigger longitudinal studies are therefore warranted to clearly delineate the effect and independent pathways of pre-treatment resistance variants detected by NGS from low frequency thresholds on treatment responses. There is also need to assess the performance of mutational frequency and absolute mutational load on treatment outcomes and to empirically determine their cut-off thresholds of clinical significance.

## Supporting information

S1 Fig Flow chart diagram illustrating PCR amplification failures despite multiple repeat attempts, from HIV-1 pre-treatment time-points 1 and 2 samples (N = 100). (DOCX)
