# Supplementary material for: Presence, persistence and effects of pre-treatment HIV-1 drug resistance variants detected using next generation sequencing: A Retrospective longitudinal study from rural coastal Kenya
Source: PLoS One. 2019 Feb 13;14(2):e0210559. doi: 10.1371/journal.pone.0210559 (PMC6373901; doi:10.1371/journal.pone.0210559)
Supplement: S1 Fig — (DOCX) [file pone.0210559.s001.docx]

Supplementary figure: Flow chart diagram illustrating PCR amplification failure, despite multiple repeat attempts, from HIV-1 pre-treatment time-point 1 and 2 samples (N=100).

Plasma samples depleted (n=7)*

Successfully amplified (n=6)

Repeat RNA extraction

Negative PCR amplification

(n=23)

Negative PCR amplification

(n=28)

Second PCR attempt

Successfully amplified (n=5)

First PCR attempt

Successfully amplified (n=72)

Pre-treatment time-points 1 & 2 samples

(n=100)

HIV-1 RNA material available

(n=16)

Third PCR attempt

Negative PCR amplification

(n=10)*

* Overall failed PCR amplification (n=17; from time-point 1 [n=2] and time-point 2 [n=15]).
